# Polymers in Sustainable Construction Composites: Rheology, Mechanical Performance, and Durability

**DOI:** 10.3390/polym17162186

**Published:** 2025-08-09

**Authors:** Yahya Kaya, Veysel Kobya, Murteda Ünverdi, Naz Mardani, Ali Mardani

**Affiliations:** 1 Department of Civil Engineering, Faculty of Engineering, Bursa Uludag University, Bursa 16059, Turkey; 512126007@ogr.uludag.edu.tr (Y.K.); vkobya@uludag.edu.tr (V.K.); murtedaunverdi@uludag.edu.tr (M.Ü.); 2 Department of Mathematics Education, Bursa Uludag University, Bursa 16059, Turkey; nazmardani@uludag.edu.tr

**Keywords:** grinding aids, TEA, TIPA, hydration kinetics, life cycle assessment

## Abstract

Today, various strategies are being adopted to produce more environmentally friendly cementitious systems. A commonly adopted strategy is the enhancement of energy efficiency in the clinker grinding process through the use of grinding aids (GAs). Another approach is to reduce cement consumption by partially replacing cement with mineral additives such as fly ash. The literature has highlighted that the use of GAs during clinker grinding can narrow the particle size distribution, thereby promoting higher rates of mineral additive replacement. Nevertheless, the literature still lacks comprehensive insight into how the combined application of commonly used GAs influences the substitution levels of mineral additives. In this regard, this study thoroughly examined the influence of varying proportions and dosages of Triethanolamine (TEA) and Triisopropanolamine (TIPA)—two commonly employed grinding aids—on the hydration kinetics, compressive strength development, and life cycle performance of fly ash (FA)-blended cementitious systems. The mixtures prepared with the cements produced were analyzed through XRD, TGA, and SEM techniques, and the compressive strength results were evaluated using the Taguchi method. The results demonstrated that, irrespective of the type of additive used, the use of GAs enhanced pozzolanic activity and compressive strength. In particular, the GA combination containing 75% TIPA and 25% TEA proved the most superior results in terms of hydration kinetics, mechanical strength, and environmental performance. It was demonstrated that the combined use of TEA and TIPA in specific proportions creates a synergistic effect, enabling the development of more efficient binder systems.

## 1. Introduction

Cement is the component that requires the highest amount of energy and cost among the materials used in cementitious systems such as concrete. This fact has led to the use of alternative mineral additives in cement production and/or in the preparation of cementitious systems, aiming for more economical and ecological production [1,2,3,4,5]. To this end, mineral additives such as fly ash (FA), ground granulated blast furnace slag, and calcined clay have been incorporated into cement production to reduce clinker content and to promote more sustainable designs [1,4,6,7]. However, these alternatives may lead to slower early strength development in cementitious systems where the clinker is partially replaced with mineral additives [4,7,8,9]. To overcome this issue and particularly to achieve higher early strength within the first 24 h of hydration, a finer grinding strategy has been adopted, focusing on increasing the proportion of particles smaller than 3 µm [7,10]. Nevertheless, producing finer-grained cements requires greater energy consumption during the clinker grinding stage. Hence, grinding aids (GAs) are employed during the clinker grinding stage to achieve a finer particle size distribution in a more energy-efficient manner. Grinding aids support the formation of fine cement particles with desired properties by reducing electrostatic forces and preventing agglomeration between particles [10,11,12,13,14,15].

Owing to their highly polar organic structures, GAs adsorb onto clinker surfaces created by the breaking of electrovalent bonds [7,13,14,15,16,17,18]. By reducing the solid surface energy and neutralizing the particle surfaces, they prevent particle agglomeration and the formation of coatings on the surfaces of grinding media, thereby creating a more efficient grinding environment [7,13,14,15,16,19].

Among the most commonly used GAs are Triethanolamine (TEA) and Triisopropanolamine (TIPA), both of which are amino alcohols with different chemical structures and hydration mechanisms. TEA primarily influences the aluminate phase and may act either as an accelerator or retarder depending on its dosage, while TIPA is known to enhance later-age strength and promote ferrite phase hydration. GAs such as TEA and TIPA can reduce the energy demand of clinker grinding by approximately 10–25%, depending on cement composition and grinding conditions [11,12]. For example, Assaad et al. [11] reported that the use of amine-based GAs led to a reduction in specific energy consumption from 45 to 34 kWh/ton in laboratory-scale grinding, while simultaneously improving Blaine fineness. These energy savings are largely attributed to reduced particle agglomeration and improved breakage efficiency during milling. Various studies have emphasized that although conventional GAs improve grinding efficiency and enhance the particle size distribution of cement, they can also lead to certain adverse effects [4,7,13,20,21,22]. For example, the effect of TEA on cement hydration remains incompletely understood, and there is no clear consensus on whether it acts as an accelerator or a retarder. When added to Portland cement at a dosage of 0.02%, TEA functions as a set accelerator, whereas at 0.25%, it exhibits a slight retarding effect. When its concentration is increased to 0.5%, the setting time is significantly prolonged, while at 1%, TEA displays a strong accelerating effect [13,23]. Although TEA is primarily known for its effect on early hydration, particularly through its interaction with the C_3_A phase, several studies have shown that it can contribute to long-term strength gains under certain dosage regimes. Similarly, TIPA, while mainly used to boost mechanical performance, has been shown to significantly accelerate hydration by enhancing the dissolution of ferrite and silicate phases, thereby contributing to increased Ca(OH)_2_ formation and bound water content [14,23,24]. TIPA may also cause incompatibility issues with water-reducing admixtures in cementitious systems [13,14]. Due to both the aforementioned negative effects observed when TEA and TIPA are used individually and the potential synergistic effects that can be achieved through their combined use, the strategy of blending TEA and TIPA in specific proportions during grinding has been proposed [25,26,27].

Mao et al. [25] investigated the effects of the combined use of TIPA/TEA GAs on the grinding and flotation behavior of quartz and reported that an appropriate dosage of a TIPA/TEA mixture (TIPA to TEA ratio of 2:1, at 0.07% dosage) improved the grinding efficiency of quartz.

Similarly, Dung and Mai [26] produced cement by using TEA and TIPA together during the clinker grinding process to enhance the early-age strength of Portland cement. Their study found that the fineness of the cement containing both TIPA and TEA was greater than that of cement containing only TEA or no additive. Moreover, at an optimum admixture dosage (0.01% TIPA + 0.02% TEA by cement weight), the setting time was shortened, accompanied by an enhancement in early-age compressive strength.

Previous research has predominantly examined the combined use of TEA and TIPA in terms of their impact on grinding efficiency and strength development. However, limited attention has been paid to how varying proportions and dosages of these additives influence hydration kinetics, compressive strength, and the life cycle performance of fly ash (FA)-blended cementitious systems. Addressing this gap, the present study investigates the effects of different TEA/TIPA combinations on FA-substituted mixtures prepared with cements produced using varying additive formulations. A comprehensive evaluation was conducted through X-ray diffraction (XRD), thermogravimetric analysis (TGA), and scanning electron microscopy (SEM) to elucidate the hydration behavior. In addition, compressive strength and life cycle assessments were performed for all mixtures, with the strength data further analyzed using the Taguchi method to identify optimal formulations. This study offers novel insights into the design of amine-based grinding aids tailored for enhanced performance and sustainability in blended cement systems.

## 2. Materials and Methods

**Figure 1:**
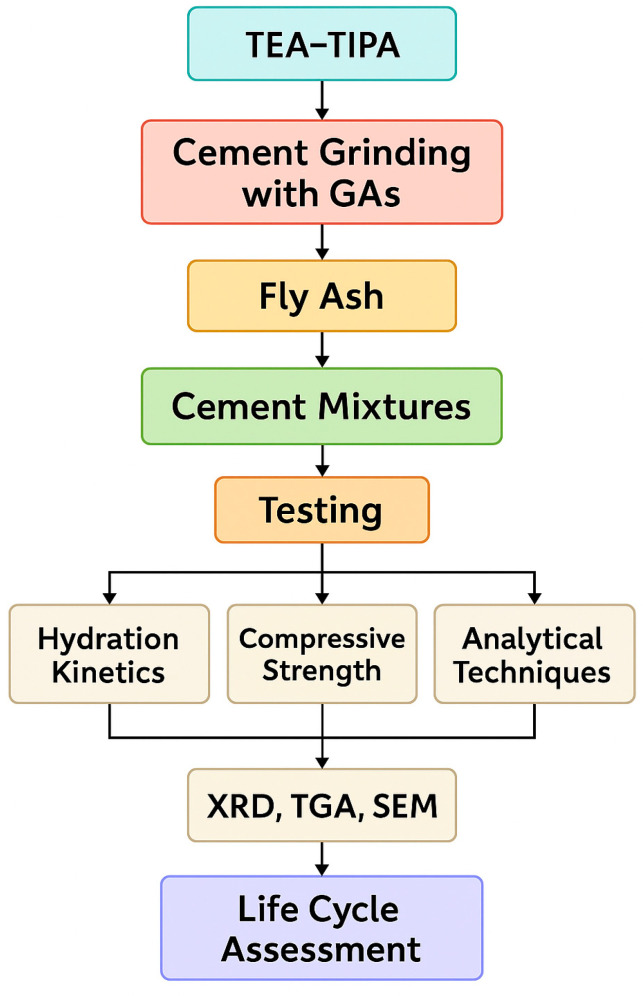
The flowchart of the study.

The flowchart of the materials and methods is shown in Figure 1.

### 2.1. Materials

In this investigation, the cement samples were fabricated by milling a blend comprising 96% clinker and 4% gypsum in a laboratory-scale ball mill until the specified Blaine surface area of 3900 ± 100 cm^2^/g was achieved. The produced cements meet the specifications for CEM I 42.5R-type cement, as outlined by the TS EN 197–1 standard [28]. In this context, some of the physical and chemical properties of the clinker, gypsum, and FA, supplied by the manufacturer, are presented in Table 1.

Table 2 presents the particle size distributions of the cements. These cements were processed to achieve a consistent Blaine surface area of 3900 ± 100 cm^2^/g following the grinding operation.

Table 2 shows that, regardless of the type of grinding aid, the particle size distributions of the cements are composed of finer particles. In this context, it can be observed that all cements containing grinding aids have a higher quantity of particles in the 3–32-micron range. This is known to be because the grinding aids adsorb onto the cement particle surface, preventing agglomeration and creating a more efficient grinding environment. The amount of particles in the 3–32-micron sieve range is directly proportional to the TIPA content. With the addition of TIPA, which positively affects the particle size distribution, a higher reaction rate in the hydration kinetics is anticipated.

**Figure 2:**
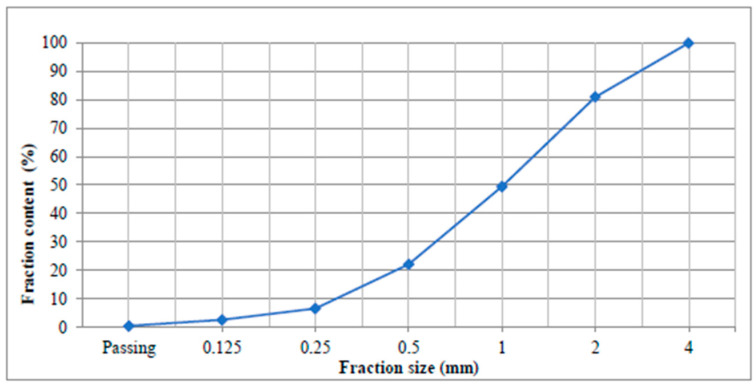
Gradation curve of the river sand aggregate used.

The preparation of the mortar mixtures utilized washed river sand aggregate, which complied with TS EN 196–1 [29] and exhibited a particle size range of 0–4 mm. The gradation curve of the aggregate is presented in Figure 2. The aggregate’s properties were evaluated per TS EN 1097–6 [30], revealing a saturated surface dry (SSD) specific gravity of 2.64 and a water absorption of 1.2%.

A uniform polycarboxylate ether-based (PCE) superplasticizer was used to achieve the target workability value in the prepared mixtures. The density, solid content, and pH value of the PCE used were measured as 1.097, 36.35%, and 3.82, respectively.

#### Grinding Aid

It is anticipated that the physical blending of different GAs could lead to a synergistic effect. To this end, both TEA and TIPA, which can enhance performance in both grinding and cementitious systems, were used. Some properties of TEA and TIPA additives are given in Table 3. In this context, five different GAs were obtained by mixing TEA and TIPA in different proportions (Table 4). The naming of the physically blended additives and the cements produced using these additives is provided in Table 4.

### 2.2. Method

#### 2.2.1. Grinding Process

**Figure 3:**
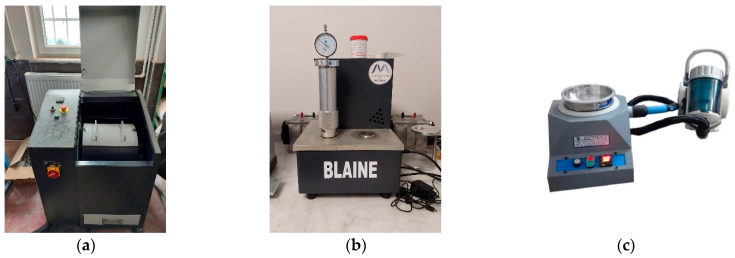
(**a**) A ball mill for material comminution; (**b**) an automatic Blaine apparatus for specific surface area measurement; and (**c**) an air jet device for particle size distribution analysis.

Clinker grinding was performed in a Bond-type laboratory mill (5 kg capacity, 1.5 kW motor) (Figure 3). Grinding continued until a Blaine fineness of 3900 ± 100 cm^2^/g was attained.

#### 2.2.2. Grinding Aid Dosages

All GA dosages were applied at 0.025%, 0.05%, and 0.1% by weight of cement, corresponding to 0.25 g, 0.5 g, and 1 g per kg of cement, respectively.

#### 2.2.3. Methodologies for the Characterization and Testing of Cementitious Materials

In this study, mortar mixtures were prepared following ASTM C109 standards [31]. All mixtures were prepared using a Hobart mixer (UTG-0130, UTEST, 2022). The flow value of the mortar mixtures was assessed by ASTM C1437 [32]. To establish the PCE requirements for the target flow, the water/binder ratio, sand/binder ratio, and target flow value were maintained constant at 0.485, 2.75, and 190 ± 20 mm, respectively. Flow measurements were conducted per ASTM C1437 [32], while the compressive strength of the mixtures was determined following ASTM C109 [31].

A number of analytical techniques were used to comprehensively investigate the effect of grinding aids (GAs) on cement hydration and microstructure. X-ray diffraction (XRD) analysis was carried out to determine the phase composition and crystal structure of the samples. Measurements were made using a diffractometer (Panalytical, Empyrean, Bursa, Turkey) with Cu-Kα radiation (λ = 1.5406 Å), a 40 kV accelerating voltage, and a 30 mA tube current. The samples were prepared by grinding into powder and sieving it through a 100-micron sieve. The scanning range was 2θ = 0–80°, with a 0.02° step width and a scanning rate of 1 s/step. Thermogravimetric analysis (TGA) was carried out to determine the thermal degradation behavior and mass losses of the samples. Measurements were made using a Perkin Elmer/Diamond thermogravimetric analyzer (Bursa, Turkey). Analyses were carried out using approximately 20 ± 2 mg of powder sample, with thermal scanning from room temperature to 1000 °C. Samples were analyzed under a nitrogen (or air) atmosphere at a heating rate of 10 °C/min. Microstructural characterization of the samples was performed using a scanning electron microscope (SEM) (Carl Zeiss/Discovery, Bursa, Turkey). In the analyses, samples taken from the cured binder systems were first dried in an oven at 50 °C for 24 h and then crushed to prepare a fine powder. A thin coating of gold/palladium (Au/Pd) was applied to the samples to increase the surface conductivity. SEM images were obtained in high-vacuum mode under an accelerating voltage of 15–20 kV. A water-to-binder ratio of 0.35 was selected for these paste mixtures, aligning with established practices in the literature [33]. At 56 days, specimens designated for XRD, TGA, and SEM analyses, specimens were carefully sectioned into small pieces (approximately 1 cm) and dipped in an isopropanol bath for two days to effectively halt cement hydration. Subsequently, these pieces were processed: a portion was ground into a powder (≤90 µm), while the remaining small pieces were kept intact. Both the powdered and small piece samples were then oven-dried at 40 °C for 48 h.

Finally, TGA and XRD analyses were conducted on the powdered samples, whereas SEM observations were performed on the small pieces.

#### 2.2.4. Designing Experiments Using the Taguchi Methodology

In this study, Analysis of Variance (ANOVA) in conjunction with the Taguchi method was employed to determine the optimal combination of grinding parameters. The Taguchi approach is based on the calculation of signal-to-noise (S/N) ratios, which can be evaluated using nominal-the-best, larger-the-better, or smaller-the-better criteria depending on the response characteristics [15]. For the present investigation, the larger-the-better criterion was adopted as the objective function, following the methodology proposed by Mandal et al. [34], as expressed in Equation (1):

(1) $$\frac{S}{N}\;=\;-10log(\frac{1}{n}\;\sum_{i=1}^{n}{1/yi}^{2})$$

Here, *yi* represents the observed data from the *i*-th experiment, and *n* denotes the number of observations.

The type of grinding aid (GA), GA dosage, and fly ash (FA) dosage were selected as the primary control factors, with their respective levels presented in Table 5. To optimize the compressive strength, splitting tensile strength, and flexural strength, as well as to evaluate the individual and interactive effects of these parameters, an L18 (6^1^ × 3^2^) orthogonal array was identified as the most suitable experimental design.

## 3. Results and Discussion

### 3.1. Hydration Kinetics

#### 3.1.1. XRD

**Figure 4:**
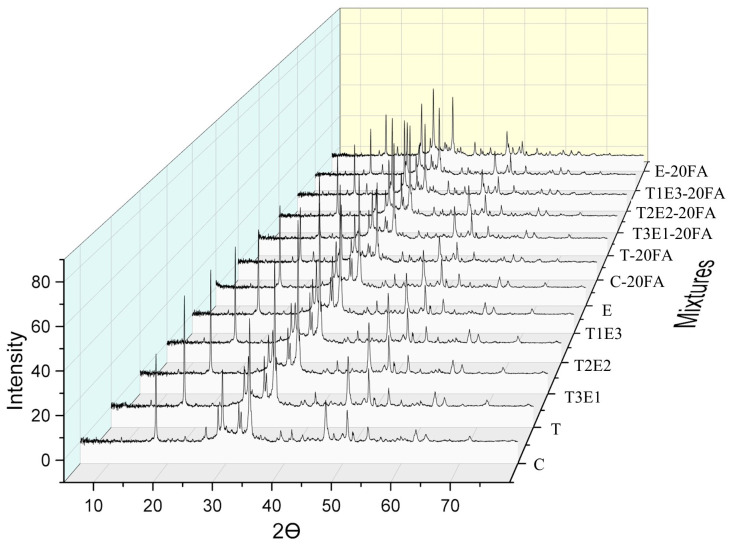
The 56-day XRD spectrum of mixtures without GA and with 0.05% GA.

Figure 4 illustrates the XRD spectra of the 56-day mixtures, comparing those prepared with cements containing 0.05% GA to a control mixture (without GA). These spectra were collected across the 0−80° 2θ range. The CH content in the 17–19° θ range is provided in Table 6. Additionally, the CH contents in the 17–19° θ range for the control, E, T, and T2E2 mixtures without fly ash, as well as the CH content for mixtures containing T, E, and fly ash, are presented in Figure 4.

In Table 6, the peak intensities of the 17–19 range (Ca(OH)_2_ spectrum) for mixtures containing and not containing pozzolan, prepared with control cement and with 0.05% GA, are given. As shown in the table, the Ca(OH)_2_ content in mixtures without supplementary cementitious materials increased by 10–30% when a GA was used, a trend observed regardless of the specific GA type. This is thought to be due to the use of GA during the clinker grinding stage. It is known that GA has both physical and chemical effects. Physically, compared to control cement, the finer particles formed with GA allow for a higher amount of Ca(OH)_2_ to be produced by increasing the hydration kinetics. In addition, depending on the GA content, TEA supports the aluminate phase, while TIPA supports the ferrite phase, which affects the production of the Ca(OH)_2_ phase. It has been determined that with the increase in the amount of TIPA, the fine particle amount also increases, thus enhancing the hydration kinetics. The literature has reported that TIPA, compared to TEA, adsorbs better onto the cement particles, leading to a finer particle size distribution [15]. Similarly to the literature, it was determined that TIPA’s physical effect on the cement particles dominates and increases the hydration kinetics, thereby supporting Ca(OH)_2_ content.

**Figure 5:**
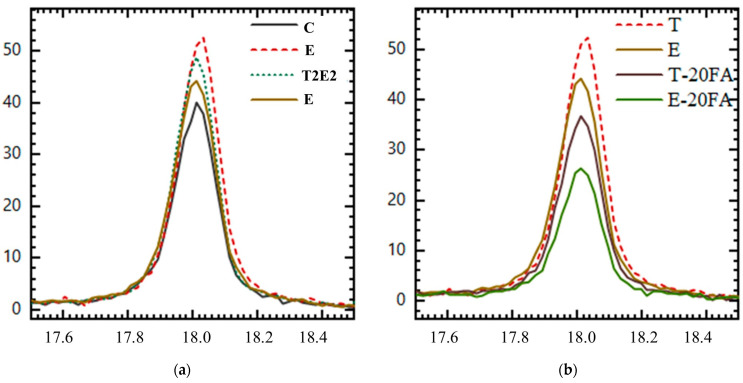
Ca(OH)_2_ peaks of (**a**) C, T, T2E2, and E mixtures; (**b**) T and E mixtures incorporating 20% fly ash.

When examining cements containing pozzolan, it is observed that the lowest and highest Ca(OH)_2_ contents are found in the E (100TEA) and T (100TIPA) mixtures, respectively. The E mixture contains a higher amount of Ca(OH)_2_ than the control mixture in the pozzolan-free mixture and a lower amount of Ca(OH)_2_ than the control mixture in the pozzolan-containing mixture. This is due to TEA significantly increasing the solubility of SiO_2_ in the presence of pozzolan, compared to TIPA. Thus, it has been determined that TEA better supports pozzolanic activity than TIPA. Chang et al. [35] reported that TEA supports aluminates in cement and slows down silicate phases, while TIPA accelerates the ferrite phase. Additionally, both additives have been reported to support pozzolanic activity by promoting the dissolution of SiO_2_ in pozzolanic systems. The Ca(OH)_2_ peaks of the C, T, T2E2, and E mixtures, as well as the T and E mixtures containing 20% FA, are presented in Figure 5.

**Figure 6:**
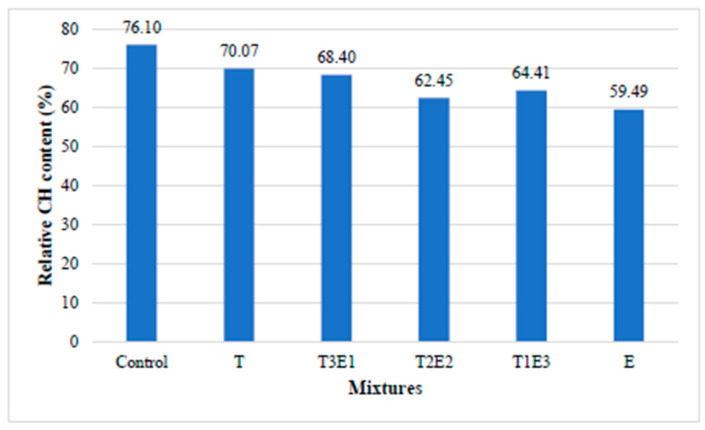
Relative Ca(OH)_2_ values of each mixture compared to their FA content.

As depicted in Figure 5a, for cementitious systems lacking fly ash, the control mixture exhibits the lowest Ca(OH)_2_ content, whereas the T mixture displays the highest. The increase in Ca(OH)_2_ content is observed to be directly proportional to the TIPA content in the GA used. As seen in Figure 5b, the Ca(OH)_2_ variation concerning FA content is presented for mixtures containing TIPA and TEA. To gain a deeper insight into the impact of GA content on pozzolanic activity, the relative Ca(OH)_2_ content percentages of each mixture, compared to the mixture prepared without FA, are shown in Figure 6.

As depicted in Figure 6, the control mixture, with the addition of 20% fly ash (FA), exhibited the lowest change in Ca(OH)_2_ content. Conversely, the E (100TEA) mixture showed the most significant change in Ca(OH)_2_ content under the same conditions. With the replacement of 20% cement by FA, a direct 20% reduction in Ca(OH)_2_ content is expected. However, it is known that the reactive SiO_2_ present in the FA, which replaces cement, reacts with the Ca(OH)_2_ formed during hydration in a pozzolanic reaction and is consumed. The consumption of Ca(OH)_2_ by the pozzolanic reaction depends on both the morphology of the Ca(OH)_2_ formed during hydration and the ability of the GAs used to ionize the Si ions in the FA. In all mixtures containing GAs, the Ca(OH)_2_ change in the presence of pozzolan was higher compared to the control mixture. This situation is related to the fact that GAs encourages the dissolution of Si ions in the FA. When focusing on the effect of TEA and TIPA in pozzolanic systems, it is observed that TEA significantly accelerates the dissolution of Si ions compared to TIPA. The literature reports that TEA and TIPA promote the dissolution of ions such as Al/Fe/Si in pozzolanic systems, thus influencing important parameters such as the Ca/Si ratio of the formed CSH gel, which significantly affects the strength compared to the control mixture without these additives [35]. Consequently, it is seen that TEA and TIPA, when used as GAs, have different mechanisms of action in pozzolanic and non-pozzolanic systems. In non-pozzolanic systems, the presence of TIPA leads to an increase in hydration kinetics, resulting in a higher Ca(OH)_2_ amount, while in the presence of pozzolan, TEA encourages the dissolution of Si, thereby increasing Ca(OH)_2_ consumption through pozzolanic reactions. This explains the optimum result achieved by using both TEA and TIPA together in pozzolanic systems.

#### 3.1.2. TGA

Literature reports indicate that the dehydration of C-S-H gel, ettringite (AFt), and monosulfoaluminate (AFm) phases causes peaks between 50 and 200 °C, which are linked to their decomposition. Peaks observed around 400–550 °C are primarily associated with the dehydration of CH [36,37]. It is important to note, however, that the partial overlap of these peaks can hinder their precise identification in TGA [38].

Given these considerations, the weight loss for the prepared mixtures was calculated within the 50–200 °C and 400–500 °C temperature ranges. As evident from the TGA curves, the CH content in the 400–550 °C range was quantified using the following equation:

(2) $$W_{\mathrm{Ca}(\mathrm{OH})2}\;=\;W_{\mathrm{loss}}\;\times \;\frac{M_{CH}}{M_{H2O}}\;\times \;100$$

In this study, W_Ca(OH)2_ denotes the calcium hydroxide (Ca(OH)_2_) content, while W_loss_ refers to the weight loss associated with Ca(OH)_2_ as identified in the TGA curves. *M_CH_* and $M_{H2O}$ correspond to the molecular weights of Ca(OH)_2_ and water, which are 74 and 18, respectively.

Non-evaporable water (NEW) represents the chemically bound water contained in the hydration products of cement, such as C-S-H and AFt phases, formed during the hydration process. In contrast to free water, which evaporates upon heating, NEW remains within the material unless it undergoes significant decomposition at elevated temperatures. This parameter plays a vital role in evaluating the degree of hydration and the long-term durability of cement-based systems [39,40]. In this study, NEW was calculated using the formula proposed by Zhang et al. [39].

(3) $$W_{n}=\frac{W1-W2\;}{W2}-\;\frac{r_{fc}}{1-r_{fc}}$$

Here, W_n_ represents the non-evaporable water content. *W*1 is the weight of the specimens at 105 °C (dried weight), and *W*2 is the weight of the specimens at 950 °C. The term rfc is a coefficient accounting for the loss on ignition (LOI) of both cement and fly FA within each mixture.

r_fc_ = p_f_ × r_f _   p_c_ × r_c_ (4)

Here, pf and pc represent the weight percentages of FA and cement, respectively, within the mixture. r_f_ and r_c_ denote the loss on ignition of FA and cement, respectively.

Table 7 presents the 56-day weight losses of FA-substituted mixtures with and without GA at a 0.05 dosage.

The existing literature indicates that NEW content can partially reflect the hydration process in pozzolan-substituted systems, similar to its role in conventional cementitious mixtures [39,40,41,42].

As seen in Table 7, the interaction with GA occurred similarly in mixtures containing FA. In the non-FA mixture with 0.05% TIPA (T), the Ca(OH)_2_ content showed a slight increase (4.5%) in comparison with the control mixture. However, this increase was eliminated in mixtures where the TEA ratio increased (T2E2, T1E3). In the non-FA mixture with 0.05% TEA (E), the Ca(OH)_2_ content decreased by 9% compared to the control.

TEA accelerates the hydration of C_3_A and C_4_AF phases while simultaneously slowing down the hydration of C_3_S [35,43,44,45]. Moreover, owing to the accelerating effect of TEA on aluminum-containing phases, its retarding effect on C_3_S becomes more pronounced [35,43]. TEA’s retardation of C_3_S hydration is associated with (i) complexing with Ca^2+^ ions, which slows down the nucleation and growth of Ca(OH)_2_ [46], and (ii) promoting the rapid formation of the TEA-AFt phase, which covers the surface of C_3_S and prevents its hydration [35].

In the non-FA T3E1 mixture, compared to the control mixture, 5.6% more Ca(OH)_2_ was observed. Here, the Ca(OH)_2_ formation in T3E1 (75% TIPA and 25% TEA) was slightly increased by 1% compared with the T mixture. This could be owing to the low TEA dosage in the mixture. Zhang et al. [47] reported that low dosages of TEA accelerate C_3_S hydration. This acceleration might stem from the rapid formation of a small amount of TEA-AFt phase on the C_3_A surface, which subsequently inhibits the growth of conventional AFt crystals, thereby facilitating faster C_3_S hydration. However, as the TEA dosage increases, this accelerating effect is replaced by a retarding effect [17]. In this study, as the TEA ratio increased, the retarding influence on the formation of the silicate phase was more dominant due to the increase in dosage.

An increase in NEW content was observed in all mixtures containing GA when compared to the control mixture. This is associated with the promotion (acceleration) of the hydration of the aluminate phase by TEA and TIPA in non-FA mixtures [35,43,44,45]. The increase in NEW in mixtures containing both GA and FA is associated with the increased pozzolanic activity of these additives. This is also evident in the decrease in Ca(OH)_2_ content in FA-containing mixtures.

**Figure 7:**
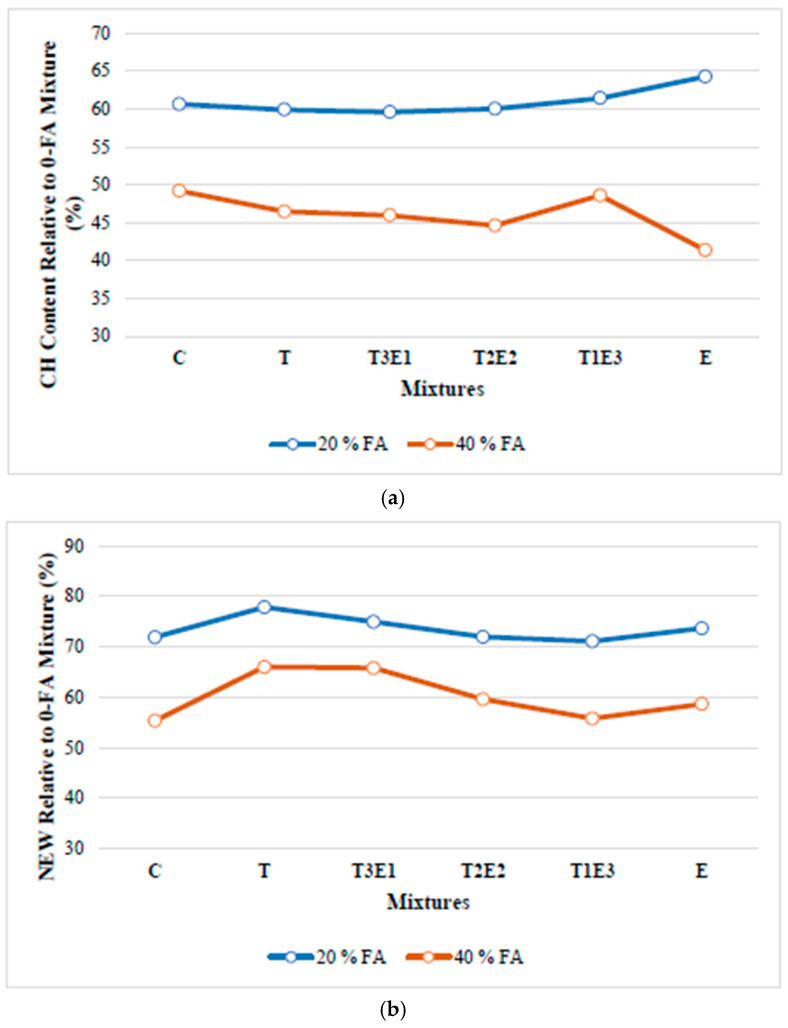
Relative (**a**) CH and (**b**) NEW contents of control and 0.05% GA-containing mixtures with respect to their counterparts without FA.

To more clearly demonstrate the changes in pozzolanic activity, the relative Ca(OH)_2_ contents and NEW of the FA-substituted mixtures, compared to the non-FA mixtures, are shown in Figure 7.

As seen in Figure 7, especially with the increase in FA replacement, a significant decrease in CH content is observed in the E mixture containing 100% TEA. This is thought to be due to the increased pozzolanic activity caused by the TEA additive. The increased pozzolanic activity of both TEA and TIPA could be due to their complexation with Ca^+2^, Al^+3^, and Fe^+3^ metals, enhancing the dissolution effect [35].

In the mixtures with 40% FA replacement, the NEW amounts of T and T3E1 mixtures are higher than those in the other mixtures containing GA. For the T mixture, this is related to the fact that with the presence of TIPA, more CH is produced compared to the other mixtures, and the increased pozzolanic activity further promotes the formation of the silicate phase in CH. There are two reasons given in the literature for TIPA’s promotion of C_3_S hydration. First, TIPA accelerates the hydration of C_4_AF, during which CH is consumed, and a suitable chemical environment may form with the CH produced by C_3_S [24,35]. Second, the increased hydration of C_4_AF exposes more surface area of the neighboring silicate phases, thereby supporting the hydration of C_3_S [48]. The observed increase for the T3E1 mixture is related to the synergistic effect formed by the combination of TEA’s pozzolanic activity-enhancing effect and TIPA.

#### 3.1.3. SEM Imaging

**Figure 8:**
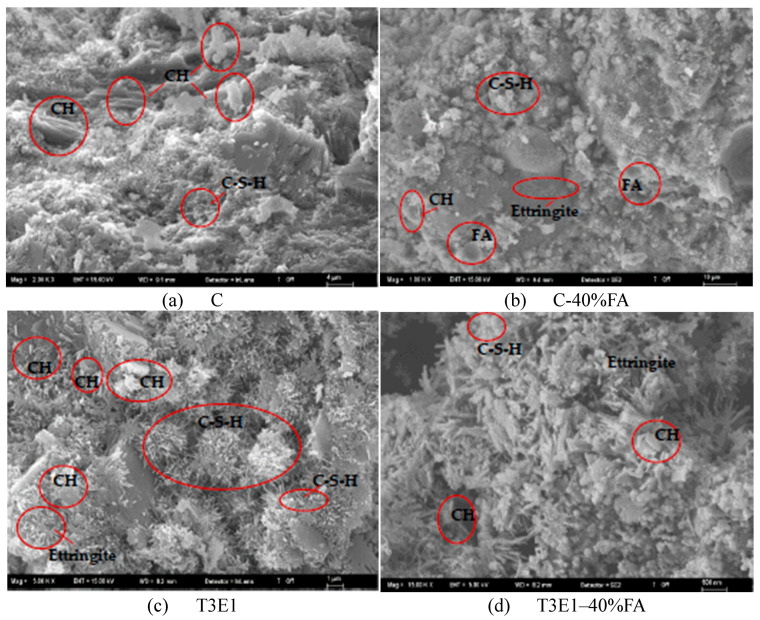

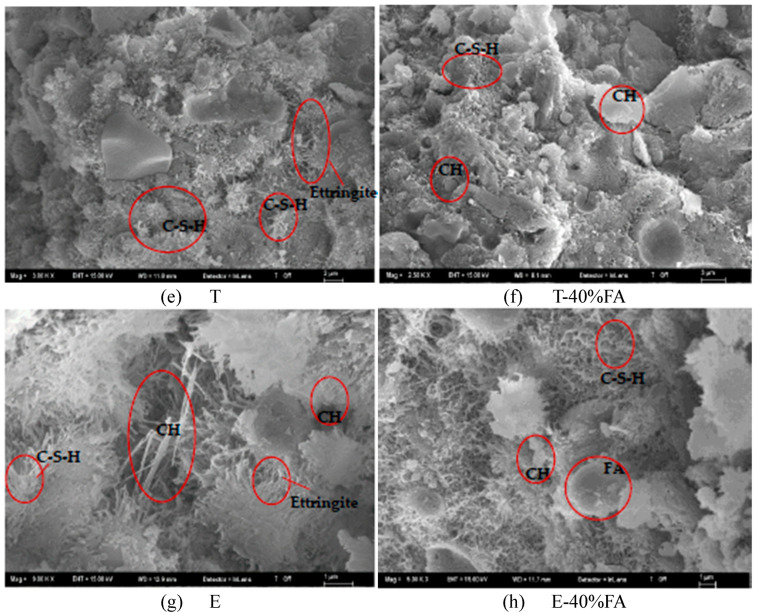
SEM images of selected mixtures: (**a**) C; (**b**) C-40%FA; (**c**) T3E1; (**d**) T3E1–40%FA; (**e**) T, (**f**) T-40%FA; (**g**) E; (**h**) E-40%FA.

The 56-day SEM images of some mixtures containing 0.05% GA and the control are shown in Figure 8. Upon examining the SEM images, mixtures containing GA with higher performance according to TGA results, such as T, T3E1, and the mixture with lower performance, E, were selected.

When examining Figure 8a,c, it is noticeable that larger Ca(OH)_2_ structures are present in the control mixture without FA. In the T3E1 mixture, however, the Ca(OH)_2_ structures have shrunk, the C-S-H phase has increased, and a more compact appearance has emerged. Similarly, in the T mixture, the C-S-H phase is more abundant compared to the control, and the overall appearance appears more void-free (Figure 8e).

In the literature, the effects of amine-based additives on C-S-H gels can be examined under three main headings [35]. The first is the direct complexation and adsorption effect of alkanolamines, which alters the shape of C-S-H gels. The second point is that alkanolamines facilitate the formation of C-S-H gels exhibiting a higher Ca/Si ratio. Thirdly, in systems incorporating TEA, the interaction with sodium silicate and saturated CH solutions leads to the disappearance of diffraction peaks on the (002) plane of the C-S-H gel during XRD analysis. This can be explained by the rearrangement of the C-S-H structure and the widening of interlayer gaps. In the SEM images, it is observed that TEA helps C-S-H gels grow more regularly, which may be related to its complexation with Ca^2+^ ions and the incorporation of TEA molecules into the gel structure. A similar behavior was observed in the SEM images obtained in this study. Additionally, alkanolamines were not added to the mixture but were used during grinding, which, as explained in the GA section, led to an increment in the amount of finer particles. The increase in fine particles also supports the increase in hydration.

In mixtures containing 40% fly ash, a decrease in pozzolanic activity compared to control mixtures (Figure 8b) is not observed in the mixtures containing GA. These mixtures appear to exhibit a higher void ratio, whereas the GA-containing fly ash mixtures (Figure 8d,f,h) display a more pronounced presence of the C–S–H phase. This observation is consistent with the TGA results presented in Table 7 and Figure 8.

### 3.2. Compressive Strength and Life Cycle Assessment

**Figure 9:**
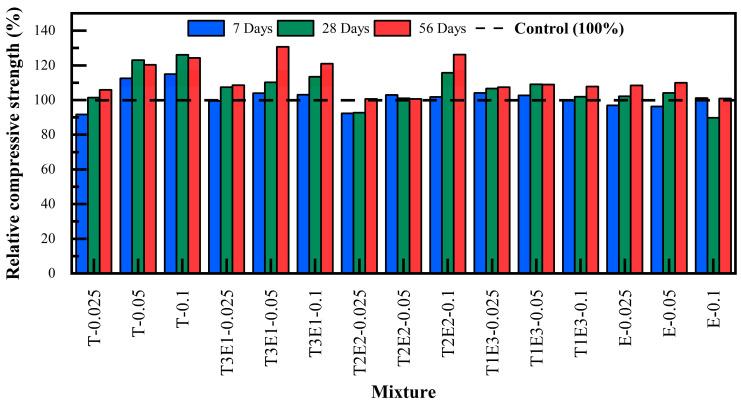
Relative compressive strength values of mixtures without FA compared to the control mixture.

**Figure A1:**
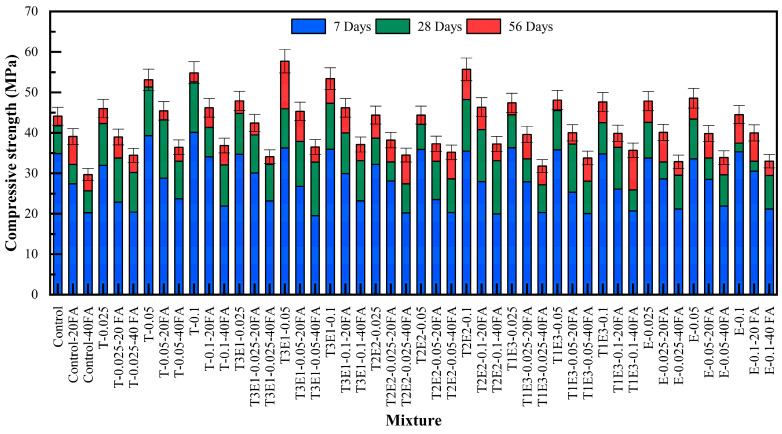
Cumulative compressive strength values for all mixtures at 7, 28, and 56 days.

Figure A1, which displays the cumulative compressive strength values for all mixtures at 7, 28, and 56 days, can be found in Appendix A. In addition, Figure 9 presents the relative compressive strength values of mixtures without FA, benchmarked against the control mixture. The GA type and dosage used in the naming of the mixtures, as well as the FA replacement ratio, were taken into account.

#### 3.2.1. Development of Compressive Strength in Cementitious Systems Without FA

In this section, the effect of the GA used in the study on the development of compressive strength in cementitious mixtures without fly ash is investigated.

As shown in Figure 9, the dosage of GAs had a direct effect on compressive strength performance. In general, increased dosage led to enhanced strength development, particularly evident in TIPA-rich systems. However, beyond a certain threshold (i.e., 0.1%), a plateauing or even slightly diminishing effect was observed in TEA-only systems, likely due to over-retardation of silicate hydration.

The mixtures with TEA (E) generally showed a positive trend in terms of long-term strength. The E-0.025 mixture showed a slight decrease of 3.1% in 7-day strength compared to the control, but no significant change was observed in 28-day strength. At 56 days, an 8.4% increase in strength was detected. Similarly, the E-0.05 mixture showed a 3.7% decrease in 7-day strength compared to the control, but a 4.1% increase at 28 days and a 10.0% increase at 56 days. In the E-0.1 mixture, there was no important distinction in 7-day strength compared to the control, but a 10% decrease in 28-day strength, and at 56 days, the strength was similar to the control.

These results are related to TEA’s acceleration of the hydration of the C_3_A phase at early ages but show that when used at high dosages, it can delay the hydration of silicates [49,50,51]. Therefore, the limited development in 28-day strength is followed by a more significant strength gain in the 56-day curing period. TEA, when used at lower dosages, does not significantly accelerate early-age hydration but maintains comparable strength levels. This can be explained by TEA’s regulatory role in the hydration kinetics of the C_3_A phase. When used at low dosages, TEA temporarily suppresses the formation of the aluminate phase by forming a stable structure with C_3_A, while contributing to the formation of sufficient C-S-H to maintain strength development [37,52,53].

The TIPA addition provided a strength increase at all ages, with particularly higher performance compared to TEA in the long term. The T-0.025 mixture showed an 8% lower performance in 7-day strength compared to the control, and did not show a significant increase in 28-day strength, but showed a 6% increase at 56 days. The T-0.05 mixture showed one of the highest improvements after 56 days with a 13% increase. The T-0.1 mixture showed a 12% increase in 56-day strength compared to the control. TIPA addition accelerates the formation of ettringite and monosulfate phases, which can lead to slower mechanical strength development within the first 7 days [54,55,56].

Looking at long-term strengths, it is believed that the simultaneous promotion of silicate and aluminate phase hydration by TIPA facilitates dissolution, contributing to more dense C-S-H gel formation, and tightening the microstructure, which are the key reasons for this improvement [57,58].

Development of Compressive Strength in Cementitious Systems Containing FA

The development of compressive strength in FA-containing systems was evaluated across multiple parameters, including curing age, FA replacement level, and the influence of GA composition, particularly the TEA/TIPA ratio. The discussion integrates both experimental results and comparative insights with the control group to understand the hydration behavior and long-term performance of blended systems.

#### 3.2.2. Effect of Curing Time

For all mixtures, compressive strength consistently increased with curing age, exhibiting the expected behavior of blended cementitious systems where hydration and pozzolanic activity develop gradually. At an early age (7 days), the reduced reactivity of FA led to significant strength loss, with mixtures such as Control-40FA showing a 42.0% decrease relative to the control. However, by 56 days, the gap narrowed substantially as pozzolanic reactions progressed, with the loss reduced to 32.7%. A similar recovery trend was observed for Control-20FA, where the 7-day loss of 21.4% diminished to 11.4% in 56 days. Similar results have been observed in some studies in the literature [59,60].

GA-containing mixtures showed superior recovery and strength development compared to the controls. For instance, T3E1–0.05, a TIPA-dominant blend, exhibited an impressive strength of 57.70 MPa at 56 days, despite its FA content, outperforming even the non-FA control. This highlights the role of GAs in enhancing hydration kinetics and accelerating the conversion of CH to secondary C–S–H gel.

#### 3.2.3. Influence of FA Replacement Level

As expected, increasing the FA content from 0% to 40% led to reduced early-age strength due to the limited initial reactivity of FA. However, this trend was mitigated in GA-modified mixtures. For example, the T-0.05–40FA mixture started with a 17.2% reduction in 7-day strength compared to the control, but ultimately showed a 24.8% increase at 56 days, clearly indicating a significant pozzolanic contribution activated by the GA.

Similarly, the E-0.05–40FA mixture exhibited a 17.5% increase in strength at 56 days despite its high FA dosage. This reflects TEA’s capacity to enhance ion dissolution and promote long-term strength development, even if its early-age influence is minimal or slightly retarding. Similar results have been obtained in some studies in the literature [50,61,62].

#### 3.2.4. Effect of GA Type and TEA/TIPA Ratio

Mixtures containing only TIPA (T-0.025 to T-0.1) achieved the highest strength levels at all ages. T-0.1 recorded 40.10 MPa (7d), 52.60 MPa (28d), and 54.84 MPa (56d), confirming TIPA’s superior impact on both aluminate and silicate phase hydration.

The T3E1 group (75% TIPA/25% TEA) also showed excellent performance, slightly trailing T at early ages but often surpassing it by 56 days. T3E1–0.05: 36.27 MPa (7d), 45.95 MPa (28d), 57.70 MPa (56d)—the highest 56-day strength across all mixtures. This indicates that the balanced combination of TEA’s early-age accelerating effect and TIPA’s later-age pozzolanic activation is effective [36,63,64,65,66].

T2E2 mixtures (50% TIPA / 50% TEA) yielded moderate early-age strength but exhibited strong long-term development. For instance, T2E2–0.1 achieved 55.70 MPa at 56 days, suitable for applications emphasizing durability over early strength.

T1E3 mixtures (25% TIPA/75% TEA) reflected TEA’s retarding impact at higher dosages. T1E3–0.1 showed slower development at 28 days but recovered well by 56 days (47.60 MPa), indicating delayed but effective pozzolanic interaction.

Mixtures containing only TEA (E-0.025 to E-0.1) recorded the lowest 28-day strengths but improved notably by 56 days. For instance, E-0.05 reached 48.55 MPa, highlighting TEA’s capacity to activate long-term pozzolanic reactions, albeit with limited early-age benefits.

Notably, hybrid mixtures like T1E3–0.05–40FA displayed a 22.3% strength increase at 56 days relative to the control, validating the benefit of blending TEA and TIPA. Overall, amino alcohol-based GAs such as TIPA and TEA significantly improved compressive strength in FA-containing systems. While TEA primarily enhances later-stage pozzolanic activity, TIPA contributes more strongly to both early and late hydration kinetics. Among all formulations, a TIPA:TEA ratio of 75:25 at a 0.05–0.1% dosage (T3E1) demonstrated the most effective performance, offering a high-strength and sustainable solution for blended cementitious systems.

#### 3.2.5. Life Cycle Assessment

This section examines the mechanical performance and environmental impacts of high levels of FA replacement in mixtures containing TIPA and TEA. The most favorable results were obtained for the mixture containing 0.1% GA (by cement weight) and 75% TIPA with 25% TEA (T3E1–0.1).

This formulation achieved a 56-day compressive strength of 53.4 MPa, showing a 20.9% increase compared to the control mixture (44.13 MPa), despite 46.1% FA replacement. This is the maximum replacement level evaluated in this study. High levels of FA replacement generally lead to strength loss at early ages and, in some cases, over the long term [58,59]. However, the strength increase observed here suggests that the selected GAs can enhance the pozzolanic activity of FA. This was also emphasized in a study by He et al. [67]. The presence of TIPA, which is known to support C_3_A hydration and late-age strength development, likely accelerates the formation of additional C–S–H, contributing to the densification of the microstructure and the maintenance of strength as the curing period extends [60].

This system also significantly impacts environmental advantages. The environmental effects and parameters, such as consumption and savings of the cement-based system mixtures in the study, were evaluated by a life cycle assessment. The consumption and energy requirements considered during the life cycle assessment are calculated according to Table 8.

When 1.4 tons of raw material is used to produce 1 ton of cement, approximately 680 kg of CO_2_ is emitted during the calcination process; when emissions from electricity and transportation are included, the total emissions reach about 950 kg [70,71]. Additionally, the production process generates 500–700 kg of industrial waste. Therefore, the use of pozzolans is being investigated as a lower-emission alternative, with CO_2_ emissions from these materials reported to be in the range of 70–100 kg per ton [68,72].

Beyond its environmental advantages, the combined use of FA and GAs significantly enhances the performance of blended cementitious systems. Grinding aids such as TEA and TIPA not only improve the energy efficiency of grinding but also modify the hydration behavior of fly ash–cement blends. Several studies have demonstrated that GAs can promote early-stage strength development by improving particle dispersion and enhancing the dissolution of active silica and alumina phases within FA, thereby accelerating pozzolanic reactions [1,24,35]. For instance, Chang et al. [35] reported that TIPA enhances ferrite phase hydration, contributing to Ca(OH)_2_ formation, which is then consumed by FA to produce secondary C–S–H gels. Similarly, Liu et al. [37] observed that GAs improve the microstructural densification in FA-blended cements, leading to improved mechanical performance at later ages. Moreover, the synergistic effect of TEA and TIPA, as confirmed in this study, can regulate both aluminate and silicate phase kinetics, thus compensating for the delayed reactivity typically associated with FA at early curing ages. Consequently, integrating FA with optimized grinding aid formulations offers not only environmental savings but also superior hydration kinetics and long-term mechanical performance compared to FA use alone.

**Figure 10:**
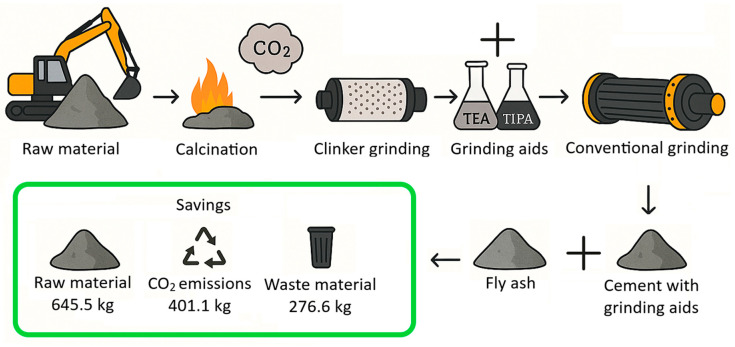
Life cycle process diagram.

**Figure 11:**
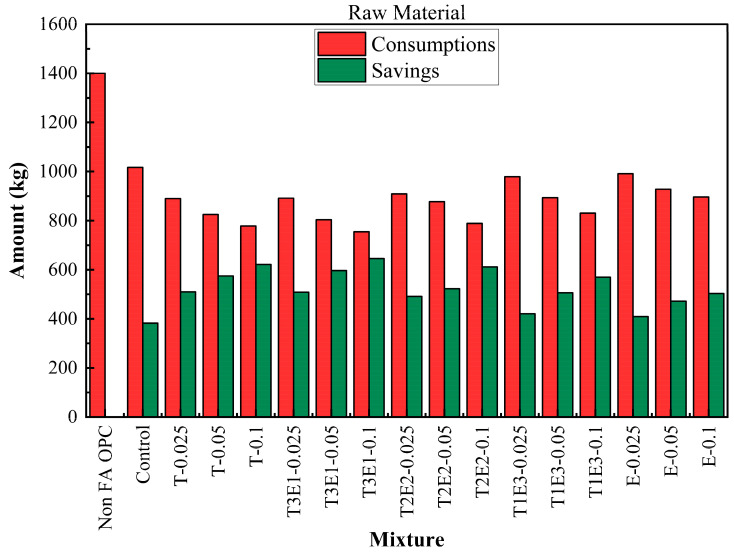
Consumptions and savings for raw materials.

**Figure 12:**
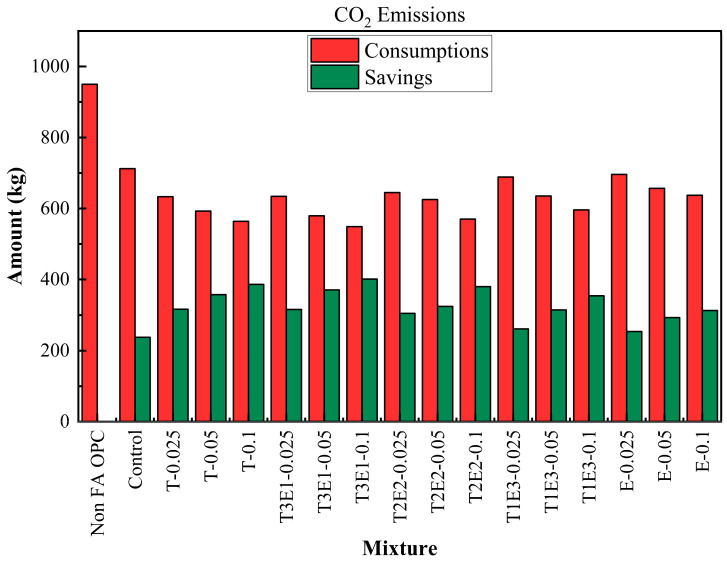
Consumption and savings for CO_2_ emissions.

**Figure 13:**
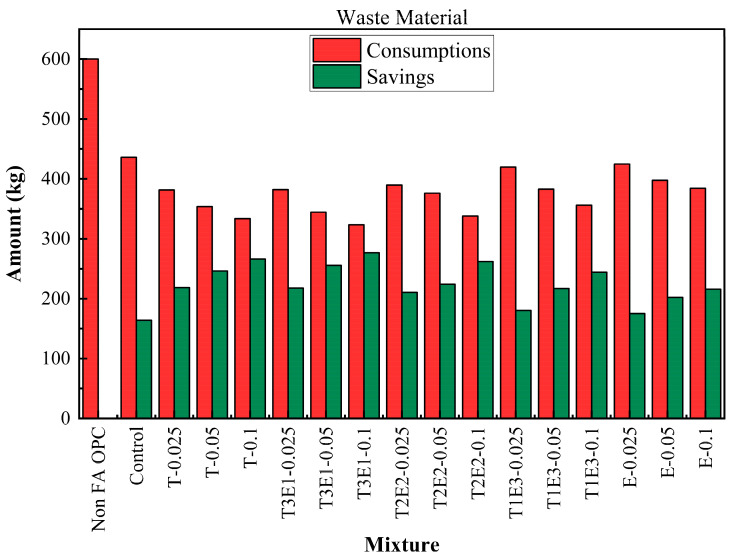
Consumptions and savings for waste material.

The life cycle process diagram for the life cycle assessment is presented in Figure 10. The consumption and savings that are determined according to the life cycle analysis, as raw material, CO_2_ emissions, and waste generation, are shown in Figure 11, Figure 12 and Figure 13. In addition, Table 9 shows the maximum FA substitution for target strength values. Here, maximum FA substitution refers to the maximum percentage of fly ash that can be applied to achieve the compressive strengths determined in the study.

Compared to the baseline non-FA OPC mixture, all other mixtures demonstrated substantial raw material savings, primarily due to the replacement of clinker with FA. The control mixture, which includes FA but no GA, already reduced consumption to 1017.45 kg, yielding a 382.55 kg saving. The integration of GAs further enhanced savings. The T3E1–0.1 mixture (75% TIPA + 25% TEA at 0.1% dosage) achieved the lowest raw material usage at 754.50 kg, marking a maximum saving of 645.50 kg. This trend confirms that synergistic use of TEA and TIPA not only promotes hydration but significantly improves material efficiency.

A similar pattern was observed in CO_2_ emissions. The reference OPC emitted 950 kg CO_2_ per ton, while the control mixture brought this down to 712.27 kg. Emission reductions became more pronounced with increasing GA effectiveness and FA content. The T3E1–0.1 mixture again recorded the lowest CO_2_ emissions (548.87 kg), offering a saving of 401.13 kg—over a 42% reduction compared to OPC. This reduction is attributed to two factors: the lower clinker demand (the major CO_2_ contributor in cement production) and the improved grinding efficiency offered by optimized TEA-TIPA blends.

Waste material savings followed the same trajectory. While the OPC mixture generated 600 kg of waste, the control dropped this to 436.05 kg. As with other metrics, T3E1–0.1 emerged as the most efficient, producing only 323.36 kg of waste and saving 276.64 kg. Other TIPA-rich or balanced TIPA-TEA combinations (e.g., T-0.1, T2E2–0.1) also showed strong reductions. These results reinforce that fly ash replacement and GA optimization not only affect strength and hydration kinetics but significantly reduce industrial waste—supporting circular economy goals.

The data highlight that the combined use of TEA and TIPA, especially in the T3E1 formulation at higher dosages, optimizes environmental performance without compromising strength. The reductions in raw material use, CO_2_ emissions, and waste generation across T3E1–0.1 reflect a high-efficiency binder system with both mechanical and ecological benefits. This synergy not only enhances pozzolanic activity but also enables deeper clinker substitution, positioning the T3E1–0.1 mixture as the most sustainable and performance-balanced option in this study.

In summary, significant savings have been achieved in the T3E1–0.1 mixture as follows:

A reduction of 645.5 kg in raw material consumption;

A reduction of 401.1 kg in CO_2_ emissions;

A reduction of 276.6 kg in solid waste generation (per ton of cement).

These gains are believed to be based on two interrelated mechanisms: (1) the partial substitution of clinker, which requires high energy and generates significant amounts of CO_2_, with FA, and (2) the increase in grinding efficiency provided by TEA/TIPA additives, reducing the energy demand of the mill and optimizing the particle size distribution [4,73]. In particular, this system supports a circular economy approach by utilizing industrial waste (FA) and reducing the environmental footprint of cement production.

Furthermore, the T3E1–0.1 mixture, with FA replacement, has been shown to exceed the strength of the control mixture, demonstrating the potential to lower the overall cement demand in concrete mix design. This has the potential to further enhance sustainability gains through material efficiency.

In conclusion, the findings suggest that the use of amino-based GAs in conjunction with high levels of FA substitution does not result in any loss of mechanical performance. On the contrary, in optimized systems such as T3E1–0.1, this approach offers a high-performance and environmentally sustainable solution.

### 3.3. Taguchi Analysis Results

#### 3.3.1. Signal-to-Noise (S/N) Ratio Analysis

The compressive strength properties corresponding to each set of control factors were determined experimentally, employing a design of experiments approach. The resulting data were subsequently processed using Taguchi techniques, and the optimization of these measured factors was accomplished through the analysis of their S/N ratios. The strength performance values with the highest value are crucial for the performance of cementitious systems and the pozzolan substitution ratio. Consequently, the “larger is better” equation was employed to calculate the S/N ratio. Table 10 presents the S/N ratios and mean values for the strength parameters.

The influence of each control factor on strength performance was evaluated using both the “S/N response table” and the “Mean table.” The corresponding results are presented in Table 11.

Prepared using the Taguchi method, this table presents the optimal levels of control factors for achieving maximum strength performance. It allows straightforward identification of the best processing parameters in cementitious systems containing GAs. For each control factor, the optimal level was selected based on the highest S/N ratio observed among its levels. Consequently, the conditions yielding the best strength performance were identified as Level 6 for factor A, Level 2 for factor B, and Level 1 for factor C.

#### 3.3.2. ANOVA Method

In this study, Analysis of Variance (ANOVA) was employed to analyze the effects of GA type, GA dosage, and fly ash dosage on the strength properties of cementitious systems. The ANOVA results for these strength properties are presented in Table 12.

The analysis was conducted at a 5% significance level and a 95% confidence level. The importance of each control factor in the ANOVA was determined by comparing its respective F-values. Furthermore, a P-value less than 0.05 for a control factor indicates its statistical significance. The final column of Table 11 displays the effect percentage, which quantifies each parameter’s contribution to the overall process performance.

The percentage contributions of GA type, GA dosage, and fly ash content to the compressive strength of cementitious systems were determined to be 11%, 5%, and 82%, respectively (Table 12). This indicates that the fly ash ratio is the most influential parameter affecting compressive strength. The ANOVA model exhibited an error percentage of 2%.

## 4. Conclusions

This study examined the effects of the combined use of TEA and TIPA, which are commonly employed in the industry, on various properties of fly ash-blended cementitious systems. The following conclusions were drawn based on the findings from hydration kinetics, compressive strength development, life cycle analysis, and optimization studies.

Regardless of the GA type, improvements in pozzolanic activity and strength were observed due to the narrowing of particle size distribution and increased hydration rates.

The combined use of TEA and TIPA at specific ratios enhanced production efficiency through a synergistic effect. Among the tested formulations, the mixture containing 75% TIPA and 25% TEA exhibited the best performance regarding hydration kinetics, compressive strength, and life cycle assessment. At this ratio, the adverse effect of TEA in delaying C-S-H formation and the air-entraining, strength-reducing effect of TIPA were effectively mitigated. In other words, the combined application of TEA and TIPA at this specific ratio overcame the limitations associated with the individual GAs, yielding synergistic benefits. Moreover, optimization studies revealed that the GA mixing ratio significantly influenced key parameters such as the optimal fly ash replacement level.

In conclusion, the findings demonstrate the promising role of GA technologies in developing next-generation, low-carbon binders. Future research should focus on evaluating these optimized blends’ long-term durability, environmental performance, and behavior under variable climatic conditions and application scenarios.

## Figures and Tables

**Table 1:** Certain characteristics of clinker, gypsum, and FA.

Item	Chemical Properties		
	Clinker	Gypsum	FA
SiO_2_	21.52	4.98	59.22
Al_2_O_3_	5.43	1.21	22.86
Fe_2_O_3_	3.31	0.83	6.31
CaO	65.38	28.94	3.09
MgO	1.04	0.83	1.31
SO_3_	0.38	39.67	0.17
Na_2_O + 0.658 K_2_O	0.83	0.37	1.4
Cl	0.01	-	0.001
C_3_S	56.51		
C_2_S	19.06		
C_3_A	8.79		
C_4_AF	10.07		
Loss on ignition	0.52		3.2
Specific surface (cm^2^/g)			4300

**Table 2:** Cement particle size distributions.

	<10 Micron (%)	10–32 Micron (%)	32–60 Micron (%)	60–100 Micron (%)	>100 Micron (%)
Control	19.52	42.02	32.42	5.42	0.62
E–0.025	22.12	42.24	30.45	4.54	0.65
E–0.05	24.42	43.00	28.12	4.04	0.42
E–0.1	25.89	44.53	25.10	3.97	0.51
T–0.025	26.53	50.09	20.20	2.20	0.98
T–0.05	27.83	51.71	17.21	2.24	1.01
T–0.1	31.38	53.92	11.46	2.49	0.75
T1E3–0.025	24.53	42.67	28.53	4.12	0.15
T1E3–0.05	25.74	43.74	25.53	4.04	0.95
T1E3–0.1	26.83	44.43	24.63	3.90	0.21
T2E2–0.025	24.96	44.12	26.14	4.24	0.54
T2E2–0.05	25.32	43.21	27.32	3.98	0.17
T2E2–0.1	26.44	44.73	23.09	4.11	1.63
T3E1–0.025	25.66	42.76	27.50	3.32	0.76
T3E1–0.05	26.23	46.31	22.73	3.24	1.49
T3E1–0.1	29.95	47.87	18.37	3.03	0.78

**Table 3:** Some properties of the TEA and TIPA admixture.

Admixture Name	Molecular Representation of Admixtures	Density (g/cm^3^)	Solid Content (%)	Chloride Content (%)	pH 25 °C	Number of Functional Groups
TEA	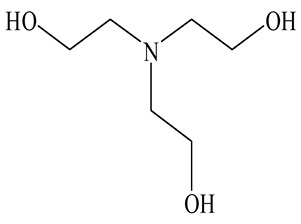	1095	50.0	<0.1	10.5	3
TIPA	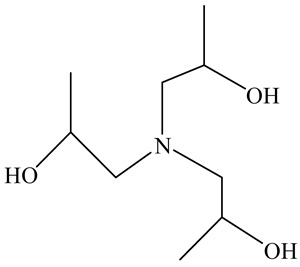	1124	50.0	<0.1	10.8	3

**Table 4:** GAs newly obtained through physical blending.

GA Type (%)	E1	T1E3	T2E2	T3E1	T1
TEA	100%	75%	50%	25%	-
TIPA	-	25%	50%	75%	100%

**Table 5:** Factor parameters and their levels.

Parameters	Symbol	Levels 1	Levels 2	Levels 3	Levels 4	Level 5	Level 6
GA Type	A	GA0	GA1	GA2	GA3	GA4	GA5
GA Dosage	B	0.025	0.05	0.1	-	-	-
FA Dosage	C	0	20	40	-	-	-

**Table 6:** Content of 17–19 range (Ca(OH)_2_ spectrum) in mixtures containing and not containing FA prepared with control cement without GA and with 0.05% GA.

	0% FA		20% FA	
	Ca(OH)_2_ Content Between 17 and 19 Ɵ	Relative Ca(OH)_2_ Compared to Control (%)	Ca(OH)_2_ Content Between 17 and 19 Ɵ	Relative Ca(OH)_2_ Compared to Control (%)
Control	40.159	100.00	30.56	100.00
T	52.56	130.88	36.83	120.51
T3E1	50.95	126.87	34.85	114.04
T2E2	48.87	121.69	30.52	99.87
T1E3	44.45	110.69	28.63	93.68
E	44.36	110.46	26.39	86.35

**Table 7:** Hydration product quantification (weight loss, Ca(OH)_2_, and NEW) in 56-day fly ash-substituted mixtures.

Mixtures	FA Content (%)	Weight Loss (wt.%)		Ca(OH)_2_ (CH) Content	NEW * (wt.%)	Relative to Control (%)	
		50–200 °C	400–550 °C			Ca(OH)_2_ CH Content	NEW
		56 days	56 days	56 days	56 days	56 days	56 days
Control	0	4.793	5.310	21.831	17.857	100.0	100.0
	20	2.994	3.221	13.242	12.840	100.0	100.0
	40	2.583	2.613	10.743	9.8850	100.0	100.0
T	0	5.126	5.549	22.814	19.282	104.5	108.0
	20	3.305	3.325	13.669	15.013	103.2	116.9
	40	2.865	2.578	10.599	12.733	98.70	128.8
T3E1	0	5.682	5.366	23.061	21.265	105.6	119.1
	20	4.348	3.343	13.743	15.935	103.8	124,1
	40	3.785	2.579	10.602	13.998	98.70	141.6
T2E2	0	5.280	5.361	22.042	21.786	101.0	122.0
	20	4.043	3.218	13.231	15.685	99.90	122.2
	40	3.348	2.393	9.8390	12.997	91.60	131.5
T1E3	0	5.986	4.773	21.622	19.522	99.00	109.3
	20	4.117	3.231	13.281	13.879	100.3	108.1
	40	2.818	2555	10.505	10.892	97.80	110.2
E	0	4.903	4.821	19.816	18.386	90.80	103.0
	20	3.794	3.099	12.742	13.545	96.20	105.5
	40	3.452	1.993	8.1950	10.789	76.30	109.1

* NEW: non-evaporated water.

**Table 8:** The consumed raw materials, resulting CO_2_ emissions, and waste quantities.

Raw Materials	Amount	Units	Reference
Limestone (soil)	2200		[68,69]
Limestone	1207	kg	[68,69]
Shale (soil)	400		[68,69]
Shale	210.4	kg	[68,69]
Gypsum (soil)	120		[68,69]
Transportation	7.05	t.km	[68,69]
Calcination			
CO_2_	680	kg	[68,69]
NO_X_	1.08	kg	[68,69]
SO_2_	0.35	kg	[68,69]
Electricity	86	kwh	[68,69]
Transportation	13.81	t.km	[68,69]
Grinding			
Electricity	41	kwh	[68,69]
Transportation	4.86	t.km	[68,69]
Waste			
Industrial	500–700	kg	[68,69]

**Table 9:** The maximum FA substitution dosages for target strength values of mixtures.

Mixture	Maximum FA (%)
Non FA OPC	0.0
Control	27.3
T–0.025	36.4
T–0.05	41.1
T–0.1	44.4
T3E1–0.025	36.3
T3E1–0.05	42.6
T3E1–0.1	46.1
T2E2–0.025	35.1
T2E2–0.05	37.3
T2E2–0.1	43.7
T1E3–0.025	30.0
T1E3–0.05	36.2
T1E3–0.1	40.7
E–0.025	29.2
E–0.05	33.7
E–0.1	36.0

**Table 10:** Experimental outcomes, signal-to-noise ratios, and mean values.

Experiment No	Control Factors			Compressive Strength (MPa)	S/N Ratio for Compressive Strength	Means for Compressive Strength
	GA Type	GA Dosage	FA			
1	GA0	0.025	0	44.1	32.8888	44.1
2	GA0	0.05	20	39.1	31.8435	39.1
3	GA0	0.1	40	29.7	29.4551	29.7
4	GA1	0.025	0	47.9	33.6067	47.9
5	GA1	0.05	20	39.9	32.0195	39.9
6	GA1	0.1	40	33.0	30.3703	33.0
7	GA2	0.025	20	39.0	31.8213	39.0
8	GA2	0.05	40	36.4	31.2220	36.4
9	GA2	0.1	0	54.8	34.7756	54.8
10	GA3	0.025	40	31.8	30.0485	31.8
11	GA3	0.05	0	48.1	33.6429	48.1
12	GA3	0.1	20	39.9	32.0195	39.9
13	GA4	0.025	20	38.2	31.6413	38.2
14	GA4	0.05	40	35.2	30.9309	35.2
15	GA4	0.1	0	55.7	34.9171	55.7
16	GA5	0.025	40	34.1	30.6551	34.1
17	GA5	0.05	0	57.7	35.2235	57.7
18	GA5	0.1	20	46.2	33.2928	46.2

**Table 11:** S/N ratios and the mean response table.

Response Table for Signal-to-Noise Ratios				Response Table for Means			
Level	GA Type	GA Dosage	FA	Level	GA Type	GA Dosage	FA
1	31.4	31.78	34.18	1	37.63	39.18	51.38
2	32	32.48	32.11	2	40.27	42.73	40.38
3	32.61	32.47	30.45	3	43.4	43.22	33.37
4	31.9			4	39.93		
5	32.5			5	43.03		
6	33.06			6	46		
Delta	1.66	0.7	3.73	Delta	8.37	4.03	18.02
Rank	2	3	1	Rank	2	3	1

**Table 12:** ANOVA results for compressive strength.

Source	Degree of Freedom (DoF)	Sum of Squares (SS)	Mean Square (MS)	F-Value	*p*-Value	Effect Ratios (%)
GA Type	5	134.61	26.922	8.58	0.005	11.15%
GA Dosage	2	58.21	29.104	9.27	0.008	4.82%
FA	2	989.67	494.834	157.65	0	81.95%
Error	8	25.11	3.139			2.08%
Total	17	1207.6				100.00%

## Data Availability

All data within the scope of the study are given in the text.

## Appendix A

Figure A1 displays the cumulative compressive strength values for all mixtures at 7, 28, and 56 days.
